# cyNeo4j: connecting Neo4j and Cytoscape

**DOI:** 10.1093/bioinformatics/btv460

**Published:** 2015-08-12

**Authors:** Georg Summer, Thomas Kelder, Keiichiro Ono, Marijana Radonjic, Stephane Heymans, Barry Demchak

**Affiliations:** 1 ^1^Center for Heart Failure Research, Cardiovascular Research Institute Maastricht (CARIM), University Hospital Maastricht, Maastricht, The Netherlands,; 2 ^2^TNO, Zeist, The Netherlands,; 3 ^3^EdgeLeap B.V., Utrecht, The Netherlands and; 4 ^4^Department of Medicine, University of California, San Diego, La Jolla, CA, USA

## Abstract

**Summary:** We developed cyNeo4j, a Cytoscape App to link Cytoscape and Neo4j databases to utilize the performance and storage capacities Neo4j offers. We implemented a Neo4j NetworkAnalyzer, ForceAtlas2 layout and Cypher component to demonstrate the possibilities a distributed setup of Cytoscape and Neo4j have.

**Availability and implementation:** The app is available from the Cytoscape App Store at http://apps.cytoscape.org/apps/cyneo4j, the Neo4j plugins at www.github.com/gsummer/cyneo4j-parent and the community and commercial editions of Neo4j can be found at http://www.neo4j.com.

**Contact:** georg.summer@gmail.com

## 1 Introduction

Network biology facilitates the understanding of complex biological systems by organizing, analyzing and visualizing knowledge and experimental data in networks. Built upon the field of graph theory, network biology provides researchers decades worth of research in the form of sophisticated graph algorithms. Software applications like Cytoscape (Saito *et* *al.*, 2012; Shannon *et* *al.*, 2003) and Gephi (Bastian *et* *al.*, 2009) are developed to provide visualization and analysis methods to data scientists, with Cytoscape being widely used in life sciences. As networks are becoming larger and more complex, the computational performance necessary to analyze them increases drastically. Moving the computation from desktop environments like Cytoscape and Gephi to powerful servers is a common method used to cope with the increasing demand for computation. We present cyNeo4j, a Cytoscape app to link Cytoscape on the desktop to a server environment using a Neo4j database. Neo4j (www.neo4j.com) is a Java-based database designed to store and query graphs. Neo4j falls in the category of NoSQL databases as it departs from the relational model used in traditional databases. Neo4j ensures transaction reliability through ACID compliance, provides a SQL-inspired query language called Cypher and its community edition is free to use and open source. Additionally, Neo4j servers can be extended using plugins to add more complex algorithms than the ones built in. CyNeo4j supports two such plugins which showcase the performance increase that can be achieved using a Neo4j and powerful computational backend. As of version 1.1, cyNeo4j supports a plugin that provides a set of network layout algorithms and a plugin that implements the Cytoscape NetworkAnalyzer. We will briefly explain how Cytoscape users can enrich their workflows with cyNeo4j and how app and algorithm developers can benefit from it.

## 2 cyNeo4j for Cytoscape users

**Fig. 1.**
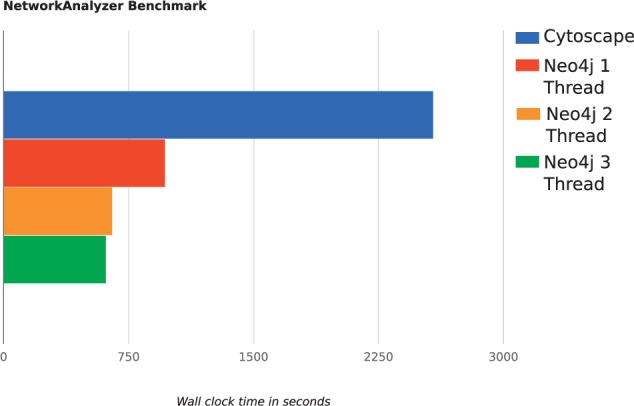
Network Analyzer Benchmark: The NetworkAnalyzer plugin implemented in Neo4j and called from cyNeo4j reduces the computation time by a factor of 4 compared to the implementation in Cytoscape. Multiple threads allow for a further decline in computation time needed. We extracted a subset of STRING with 4436 nodes and 93 286 edges

A prerequisite for cyNeo4j is a running Neo4j server. Neo4j provides thorough documentation to setup the server, additionally tutorials are available on the cyNeo4j website. The server can be run on the same computer or ideally on a computationally more powerful one. The first task for a Cytoscape user is to connect to a Neo4j database. After the connection is established and validated by cyNeo4j, the app discovers all algorithms available on the server and supported by the app itself. There are two typical use-cases for cyNeo4j: the network to be analyzed is available locally in Cytoscape or a network is stored in the running Neo4j server. In the first case the user can upload a network from Cytoscape to the Neo4j server and then run algorithms on it both locally and on the Neo4j server. This allows for an interactive workflow that uses the computational strength of the Neo4j server without interrupting the normal workflow in Cytoscape. Figure 1 shows the results of a benchmark for the NetworkAnalyzer (Assenov *et* *al.*, 2008) functionality present in Cytoscape. The Neo4j implementation cuts the waiting time for the network statistics by a factor of 4 in a subset of the STRING network (Szklarczyk *et* *al.*, 2014) with 4436 nodes and 93 286 edges if run on the same computer, producing the same statistical results (disregarding rounding behaviour). A Dell XPS 2015 (8 GB RAM, SSD, Intel Core i7-5500U 2.4 GHz) was used to compute both the Cytoscape and cyNeo4j NetworkAnalyzer results. The second use case envisions a network already stored on the Neo4j server. This network can be larger than one that is feasible to work with in Cytoscape. While it might not be possible to have the whole network locally, Neo4j is a fully fledged database and parts can be extracted using the Cypher query language for targeted analysis. Algorithms in Neo4j can still be executed on the larger network and the results then studied in smaller chunks accessed through Cypher. Currently two example algorithms are implemented as Neo4j plugins to showcase the cyNeo4j app.

1. The NetworkAnalyzer plugin for Neo4j calculates network statistics (e.g. betweenness centrality, average shortest paths, etc.) similar to the one shipped in Cytoscape. The resulting statistics are added as properties to the local Cytoscape network and can be used for visualization using the standard Cytoscape VizMapper functionality. Additionally, the statistics can be saved in the Neo4j network.
2. The ForceAtlas2 (Jacomy *et* *al.*, 2014) plugin brings a layout algorithm for large graphs from Gephi to Cytoscape via Neo4j. CyNeo4j allows the user to execute this layout with the same parameters as in Gephi and in a similar iterative and interactive fashion.

## 3 cyNeo4j for Developers

This section will give a short overview on how to implement algorithms as Neo4j plugins and how to integrate them in cyNeo4j.

### 3.1 Implementation of algorithms in Neo4j

On the server side, plugins have access to the complete Neo4j Java API including a set of graph algorithms tailored for Neo4j and the query language Cypher. These plugins can vary widely in how Neo4j capabilities are utilized: The Neo4j NetworkAnalyzer plugin heavily depends on the shortest path and centralities algorithms of Neo4j, whereas the ForceAtlas2 algorithm only uses the nodes and edges retrieval functionality of the API. Third party libraries can also be used to add functionality. The standard plugin interface of Neo4j allows plugins to return sets of basic variable types (numerics and strings).

### 3.2 Extension of cyNeo4j with new algorithms

CyNeo4j uses the Cypher and Plugin REST API through HTTP to communicate with Neo4j. New algorithms can be added to Neo4j as plugins which can be accessed through the REST API of the server making them easy to reuse in web applications or data analysis environments like R. New Neo4j plugins need to be added to cyNeo4j to allow proper discovery upon connecting to the Neo4j server, integration into the Cytoscape UI and interpretation of the algorithm results. The integration into cyNeo4j also allows for an iterative execution of an algorithm by sending multiple requests to the server enabling the user to interrupt the execution or observe intermediate results as shown with the ForceAtlas2 plugin. Algorithms can be executed asynchronously to not block Cytoscape during long running calculations. In this case the user has to determine when the calculation is done and has to retrieve the results manually.

## 4 Conclusion

We developed cyNeo4j to connect Cytoscape and Neo4j allowing us to speed up the performance of network analysis algorithms and use the Cypher query language to navigate and explore networks too large for typical desktop computers.

## Funding

The project was supported through the Google Summer of Code 2014 and the European Union (FP7-HEALTH-2010), MEDIA, large-scale integrating project grants.

*Conflict of Interest*: none declared.
